# Supplementary material for: Rapid Assessment of Susceptibility of Bacteria and Erythrocytes to Antimicrobial Peptides by Single-Cell Impedance Cytometry
Source: ACS Sens. 2023 Jul 8;8(7):2572–82. doi: 10.1021/acssensors.3c00256 (PMC10391704; doi:10.1021/acssensors.3c00256)
Supplement: Supplementary file 1 — se3c00256_si_001.pdf [file se3c00256_si_001.pdf]

Supporting Information

Rapid assessment of susceptibility of bacteria and erythrocytes to antimicrobial peptides by single-cell impedance cytometry

Cassandra Troiano[a,‡], Adele De Ninno[b,‡], Bruno Casciaro[c], Francesco Riccitelli[a], Yoonkyung Park[d], Luca Businaro[b], Renato Massoud[e], Maria Luisa Mangoni[c], Paolo Bisegna[f], Lorenzo Stella[a,*], Federica Caselli[f,*].

[a] Department of Chemical Science and Technologies, University of Rome Tor Vergata, 00133 Rome, Italy

[b] Institute for Photonics and Nanotechnologies, Italian National Research Council, 00133 Rome, Italy

[c] Laboratory affiliated to Pasteur Italia-Fondazione Cenci Bolognetti, Department of Biochemical Sciences "A. Rossi Fanelli", Sapienza University of Rome, 00185 Rome, Italy

[d] Department of Biomedical Science, College of Natural science, Chosun University, Gwangju 61452, Republic of Korea

[e] Department of Experimental Medicine, University of Rome Tor Vergata, 00133 Rome, Italy

[f] Department of Civil Engineering and Computer Science, University of Rome Tor Vergata, 00133 Rome, Italy

(‡, equal contribution; *, corresponding authors)

Emails: L. Stella, stella@stc.uniroma2.it; F. Caselli, caselli@ing.uniroma2.it

	**Spencer et al. 2020** [1]	**Tang et al. 2022** [2]	**Tang et al. 2023** [3]	**Present work**
	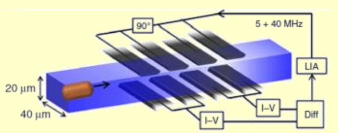	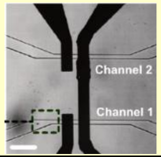	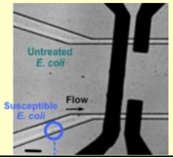	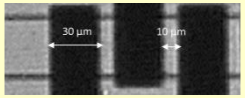
**Channel dimension (w x h)**	40 µm × 20 µm	10 µm × 10 µm	10 µm × 10 µm	40 µm × 20 µm
**Electrode layout**	4 pairs of facing electrodes	3 coplanar electrodes arranged in 2 parallel channels	3 coplanar electrodes arranged in 2 parallel channels	3 coplanar electrodes
**Electrode dimension**	30-µm wide with 10-µm gaps	30-µm wide with 15-µm gaps	30-µm wide with 15-µm gaps	30-µm wide with 10-µm gaps
**Measurement scheme**	measuring scheme using phase shifted voltages to reduce baseline current	conventional differential measurement scheme	conventional differential measurement scheme	conventional differential measurement scheme
**Frequencies**	2 simultaneous frequencies: 5 & 40 MHz	2 simultaneous frequencies: 0.5 & 6 MHz	2 simultaneous frequencies: 0.5 & 3 MHz	2 simultaneous frequencies: 0.5 & 10 MHz, or 0.5 & 20 MHz
**Electrical metrics**	electrical diameter and phase	electrical diameter and phase	electrical diameter, phase, opacity, tilt	electrical diameter and phase
**Sample type**	different strains of *K. pneumoniae*, *E. coli*, *S. aureus*, *A. baumannii*, and *P. aeruginosa*, spiked with 1.5 µm polystyrene beads	*E. coli*; polystyrene beads (500, 750 nm, 1, 2, 3, 4.5 µm, and a mixture of 3 and 4.5 µm)	*E. coli*; polystyrene beads (3 and 4.5 µm, either separately or in a mixture)	*B. megaterium*, RBCs, either separately or in a mixture, spiked with 4.5 µm polystyrene beads
**Sample treatment**	incubation (30 min at 37 °C) with Meropenem at different concentrations (0-8 mg/L range); exposure to antibiotics with different modes of action at clinical breakpoint	treatment with Mecillinam (1 µg/mL for 8 h) followed by cell fixing (70% ethanol at 23 °C)	treatment with Mecillinam (2 µg/mL for 6 h) followed by cell fixing (70% ethanol at 23 °C)	incubation (30 min at 37 °C) with DNS-PMAP23 antimicrobial peptide at different concentrations (0-2 µM range)
**Acquisition throughput**	approximately 10^5 cells in 2–3 min	n.a.	more than one thousand cells per min	a hundred per second
**Signal processing approach**	commercial lock-in amplifier and custom software written in MATLAB	lab-made lock-in amplifier and real-time signal visualization	lab-made lock-in amplifier and real-time processing in MATLAB and Labview; machine learning-based workflow enabling online training	commercial lock-in amplifier and custom software written in MATLAB
**Main features/results**	rapid (< 1 h) AST; the measured electrical characteristics reflect the phenotypic response of the bacteria to the mode of action of a particular antibiotic	simultaneous detection of reference and target particles in two separate microchannels; discrimination of susceptible and insusceptible cells in the target suspension	online training method on reference particles; real-time analysis of the proportion of susceptible cells within heterogeneous populations	rapid (< 1 h) assessment of susceptibility of *B. megaterium* and RBCs to a representative AMP; proof-of-concept application to a mixed sample

Table S1: Literature survey of impedance-based systems for susceptibility assessment of bacteria at the single-cell level (AST, antimicrobial susceptibility test; AMP, antimicrobial peptide; n.a., not available). Image snapshots are adapted with permission from: ref.[1] copyright 2020 Author(s), ref.[2] copyright 2022 American Chemical Society, ref.[3] copyright 2022 Elsevier B.V.

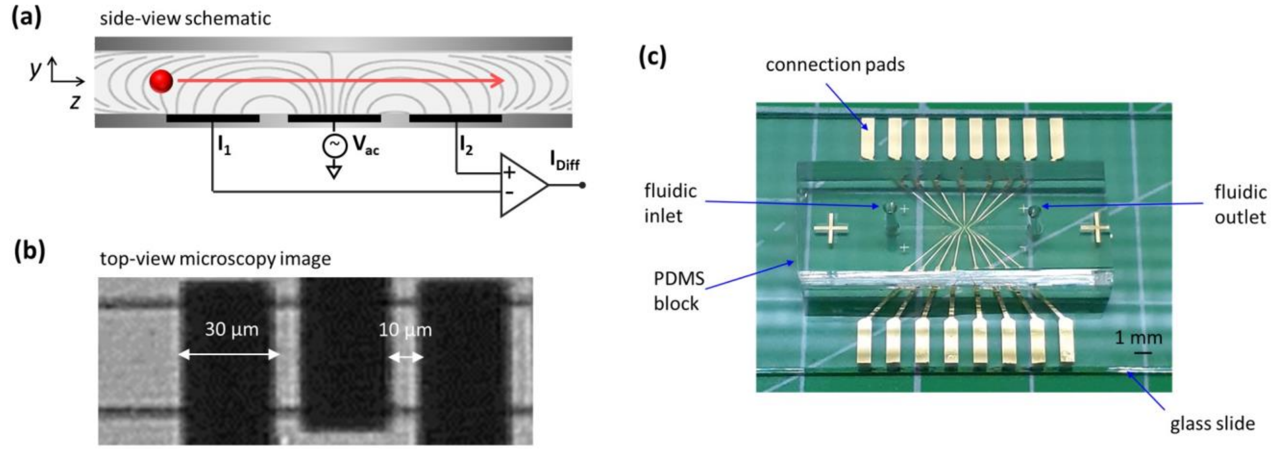

Figure S1. (a) Schematic representation of the coplanar-electrode microfluidic impedance cytometer (side view): AC excitation signals (V_{ac}) at different stimulation frequencies are simultaneously applied to the central electrode, and the difference in current flowing through the lateral electrodes is measured, $I_{Diff} = I_2 - I_1$. (b) Microscopy image of the electrical sensing zone (top view). Dark bands denote electrodes. Relevant dimensions are indicated. (c) Image of the microfluidic impedance chip. The chip is made of a PDMS block containing the microfluidic channel, bonded to a glass slide with deposited Ti/Au microelectrodes. The pads for the electric connections and the fluidic access ports are indicated in the picture.

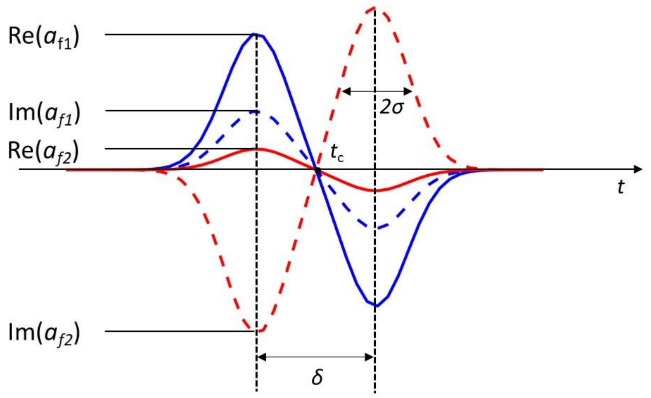

Figure S2. Bipolar Gaussian template used to fit the single-cell events:

$$s_f(t) = a_f\left(e^{-\frac{(t-(t_c-\delta/2))^2}{2\sigma^2}} - e^{-\frac{(t-(t_c+\delta/2))^2}{2\sigma^2}}\right) \qquad (1)$$

The template is characterized by the complex frequency-dependent amplitude a_f, the peak-width control σ, the peak-to-peak time δ and the central time t_c. The complex amplitude a_f is converted in modulus and phase. Since at low frequencies signal amplitude is proportional to cell volume, its cube root provides an electrical measure of the cell diameter (the electrical diameter).

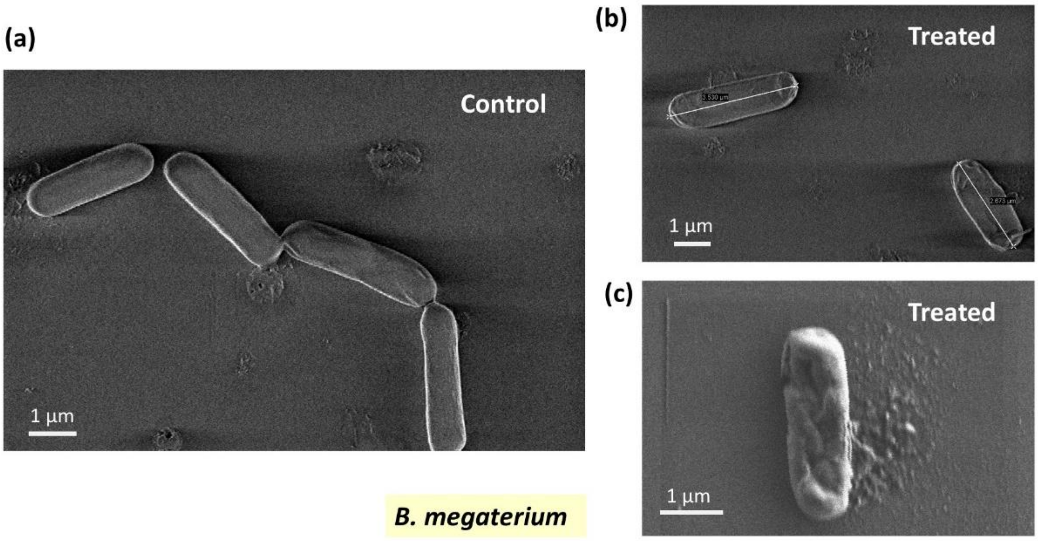

Figure S3. Scanning Electron Microscopy (SEM) images of *Bacillus megaterium* cells. *B. megaterium* (4×10^5 CFU/mL) in buffer A (5 mM HEPES, pH=7.3, 110 mM KCl, 15 mM glucose) was treated with 2 µM of DNS-PMAP23 for 30 minutes at 37 °C and then centrifuged for 10 minutes at 12000 × g. The pellet was washed with buffer A and centrifuged for 10 minutes at 12000 × g. The cells were fixed with 2.5% (v/v) glutaraldehyde in buffer A for 2 hours and subsequently centrifuged for 10 minutes at 12000 × g and washed with buffer A. The cells were then deposited on poly(L-lysine)-coated glass slides (25 × 75 × 1 mm) and dehydrated with a graded ethanol series. All bacteria specimens were visualized under high vacuum in a Zeiss Sigma 300 FEG-SEM (Field Emission Gun-Scanning Electron Microscope). Images were collected using a secondary Everhart–Thornleye detector and inlens detector at 0.9-1 kV, with a 3-5 mm working distance and a 10-15 µm objective lens aperture.

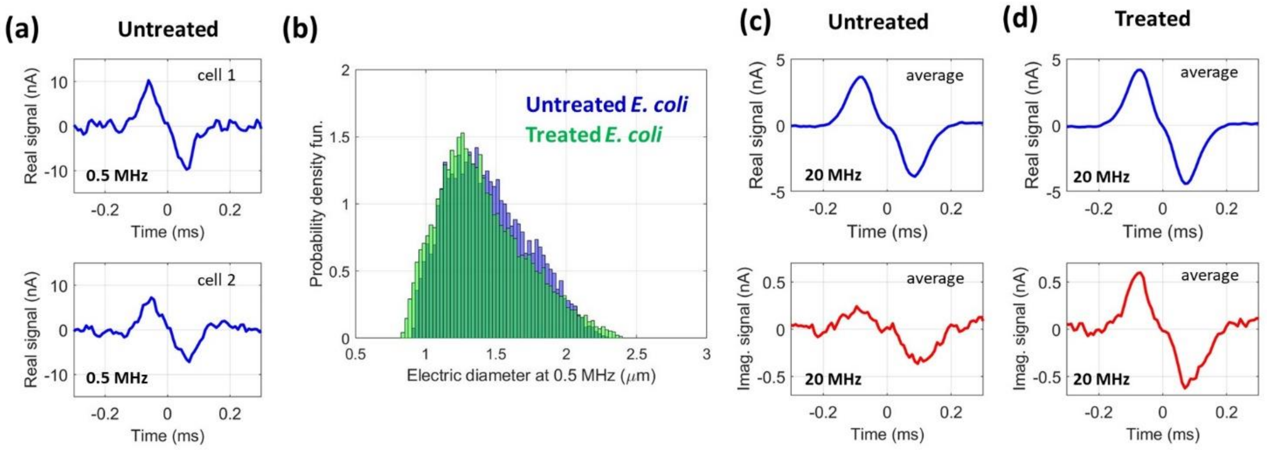

Figure S4. Impedance-based characterization of *Escherichia coli* cells. To test the sensitivity of our impedance cytometry system, additional experiments were carried out with *Escherichia coli (E. coli)* bacterial cells, which are Gram-negative rod-shaped cells with a size of about 2 µm in length and 1 µm in diameter. *E. coli* (wild type, ATCC 25922,) was grown in LB (Luria−Bertani broth) medium at 37 °C in an orbital shaker until a mid-log phase was reached, as indicated by absorbance at 590 nm of 0.8. Bacterial cells were centrifuged (1400 × g for 10 min, Eppendorf 5702 centrifuge, Hamburg, Germany) and washed eight times in buffer A (5 mM HEPES, pH=7.3, 110 mM KCl, 15 mM glucose), to remove traces of LB medium. Prior to impedance measurements, bacterial cells were diluted in buffer A to a final cell density of 4×10^5 CFU/mL. An aliquot of the bacterial suspension was incubated with DNS-PMAP23 at 2.5 µM at 37 °C for 30 minutes (treated sample). Panel (a) shows two examples of the recorded impedance signals (real part at 0.5 MHz, from the untreated sample), showing a reasonable signal-to-noise-ratio (SNR) despite the small size of the bacterial cells. Panel (b) compares the histograms of the electric diameter at 0.5 MHz for the untreated and treated samples. Mean values ± std turned out to be 1.44 ± 0.29 µm and 1.40 ± 0.31 µm, respectively. Whilst *E. coli* sphering and volume change have been reported after 8 hours incubation with 1µg/mL Mecillinam[2], no significant change of the electrical size was found after 30 min incubation with DNS-PMAP23. Acquisitions at 20 MHz were also carried out, which exhibited a lower SNR than the 0.5 MHz signals. To increase the SNR, the single-cell signals were registered and averaged before the feature extraction, as usually done when recording evoked potentials in the brain. Panel (c) [resp. (d)] shows the real and imaginary part of the average event signal at 20 MHz for the untreated [resp. treated] sample. The corresponding electric phases - normalized with respect to the phase of 3 µm diameter reference beads - turned out to be -0.2 rad (untreated sample) and -0.1 rad (treated sample). This preliminary result encourages the use of the high-frequency phase as a metric to quantify the effect of the peptide treatment. Further studies will be dedicated to optimizing the experimental setup to increase the SNR (e.g., by reducing the channel height from 20 to 15 or10 µm, and via frequency optimization).

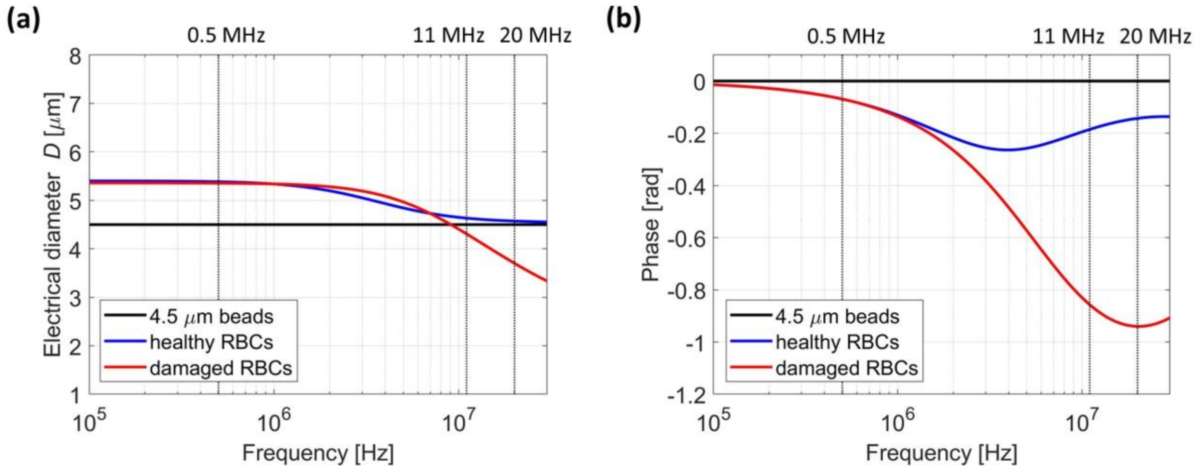

Figure S5. Simulated impedance spectra for beads, healthy RBCs, and damaged RBCs, based on Maxwell's mixture theory and the single-shell model (cf. e.g. Refs. [4,5] for the relevant theory). The electrical diameter and the phase are shown in panel (a) and (b), respectively. The three experimental frequencies (0.5 MHz, 11 MHz, and 20 MHz) are highlighted. Simulation parameters are collected in **Table S2** below. Beads and healthy RBCs parameter values are taken from Refs. [5,6]. Buffer conductivity and relative permittivity are relevant to PBS. To mimick peptide-induced pore formation, the damaged RBCs have higher membrane conductance than healthy RBCs. Moreover, damaged RBCs interior parameters are set assuming that 80% of the cytoplasm is replaced by the buffer. This model explains the lower high-frequency phase (and the lower electrical diameter at 20 MHz) exhibited by damaged RBCs with respect to healthy RBCs.

Table S2. Simulation parameters.

**Particle/medium**	**Diameter [μm]**	**Interior conductivity [S/m]**	**Interior relative permittivity [-]**	**Membrane capacitance [mF/m^2]**	**Membrane conductance [S/m^2]**
4.5 μm beads	4.5	2.7×10^{-3}	2.5	-	-
Healthy RBCs	5.4	0.52	65	8.89	10^3
Damaged RBCs	5.4	1.4	77	8.89	10^4
Buffer	-	1.6	80	-	-

References

(1) Spencer, D. C.; Paton, T. F.; Mulroney, K. T.; Inglis, T. J. J.; Sutton, J. M.; Morgan, H. A Fast Impedance-Based Antimicrobial Susceptibility Test. *Nat. Commun.* **2020**, *11* (1). https://doi.org/10.1038/s41467-020-18902-x.

(2) Tang, T.; Liu, X.; Yuan, Y.; Zhang, T.; Kiya, R.; Yang, Y.; Yamazaki, Y.; Kamikubo, H.; Tanaka, Y.; Li, M.; Hosokawa, Y.; Yalikun, Y. Parallel Impedance Cytometry for Real-Time Screening of Bacterial Single Cells from Nano- to Microscale. *ACS Sensors* **2022**, *7* (12), 3700–3709. https://doi.org/10.1021/acssensors.2c01351.

(3) Tang, T.; Liu, X.; Yuan, Y.; Kiya, R.; Zhang, T.; Yang, Y.; Suetsugu, S.; Yamazaki, Y.; Ota, N.; Yamamoto, K.; Kamikubo, H.; Tanaka, Y.; Li, M.; Hosokawa, Y.; Yalikun, Y. Machine Learning-Based Impedance System for Real-Time Recognition of Antibiotic-Susceptible Bacteria with Parallel Cytometry. *Sens. Actuator B-Chem.* **2023**, *374*, 132698. https://doi.org/10.1016/j.snb.2022.132698.

(4) Honrado, C.; Bisegna, P.; Swami, N. S.; Caselli, F. Single-Cell Microfluidic Impedance Cytometry: From Raw Signals to Cell Phenotypes Using Data Analytics. *Lab Chip* **2021**, *21* (1), 22–54. https://doi.org/10.1039/D0LC00840K.

(5) De Ninno, A.; Reale, R.; Giovinazzo, A.; Bertani, F. R.; Businaro, L.; Bisegna, P.; Matteucci, C.; Caselli, F. High-Throughput Label-Free Characterization of Viable, Necrotic and Apoptotic Human Lymphoma Cells in a Coplanar-Electrode Microfluidic Impedance Chip. *Biosens. Bioelectron.* **2020**, *150*, 111887. https://doi.org/10.1016/j.bios.2019.111887.

(6) Spencer, D.; Morgan, H. High-Speed Single-Cell Dielectric Spectroscopy. *ACS Sensors* **2020**, *5* (2), 423–430. https://doi.org/10.1021/acssensors.9b02119.
